# A cohort study of factors that influence oral health-related quality of life from age 12 to 18 in Hong Kong

**DOI:** 10.1186/s12955-020-01317-z

**Published:** 2020-03-10

**Authors:** Ling Sun, Hai Ming Wong, Colman P. J. McGrath

**Affiliations:** 1 Paediatric Dentistry and Orthodontics, Faculty of Dentistry, The University of Hong Kong, 2/F, Prince Philip Dental Hospital, 34 Hospital Road, Sai Ying Pun, Hong Kong SAR, China; 2 Department of Orthodontics, Stomatological Centre, Guangzhou Women and Children’s Medical Centre, Guangzhou, China; 3 Periodontology and Public Health, Faculty of Dentistry, The University of Hong Kong, Hong Kong SAR, China

**Keywords:** Oral health-related quality of life, Periodontal status, Caries, Malocclusion, Sociodemographic factors

## Abstract

**Background:**

There is a lack of cohort studies on the influence factors of oral health-related quality of life (OHRQoL). This study aimed to follow subjects from age 12 to 18 to analyse the sociodemographic and clinical factors that may influence OHRQoL.

**Methods:**

This cohort study selected a representative sample from Hong Kong. Periodontal status and caries were examined according to WHO criteria. Four orthodontic indices were used to assess malocclusion. Child Perceptions Questionnaires (CPQ_11–14_) with 8 items (CPQ_11–14_-ISF: 8) and 37 items were used to assess OHRQoL at age 12 and age 15, respectively; Oral Health Impact Profile (OHIP-14) was used to assess OHRQoL at age 18. Wilcoxon signed ranks test and Friedman’s test were used to analyse the age-related change of OHRQoL and malocclusion from age 12 to 18. Generalized estimating equations were used to analyse the influence factors of OHRQoL and to calculate adjusted risk ratio (RR).

**Results:**

Subjects recruited in this study were 589 (305 females, 284 males), 364 (186 females, 178 males) and 300 (165 females, 135 males) at age 12, 15 and 18, respectively. Among them, 331 subjects (172 females, 159 males) were followed from age 12 to 15, and 118 subjects (106 females, 82 males) were followed from age 12 to 18. Subjects had less severe malocclusion at age 12 than at ages 15 and 18 (*p* = 0.000, measured by Dental Aesthetic Index). Age, periodontal status, and malocclusion had an effect on OHRQoL. When compared with OHRQoL at age 12, worse OHRQoL was observed at age 15 (adjusted RR = 1.06, 95%CI = 1.01–1.12, *p* = 0.032), but not at age 18 (adjusted RR = 1.01, 95%CI = 0.95–1.08, *p* = 0.759). Unhealthy periodontal conditions had a negative effect on OHRQoL (adjusted RR = 1.14, 95%CI = 1.04–1.25, *p* = 0.007). Only severe malocclusions had a negative effect on OHRQoL; a more severe malocclusion was associated with a higher effect on OHRQoL (adjusted RR = 1.09, 95%CI = 1.01–1.18, *p* = 0.032 for severe malocclusion, and adjusted RR = 1.17, 95%CI = 1.07–1.28, *p* = 0.001 for very severe malocclusion measured by Dental Aesthetic Index).

**Conclusion:**

Age, periodontal status, and malocclusion had an influence on OHRQoL from age 12 to 18. When clinicians attempt to improve subjects’ OHRQoL, it is necessary to consider these factors.

## Introduction

The psychosocial aspects of dentistry, such as dental fear, treatment satisfaction and oral health-related quality of life (OHRQoL), have been increasingly drawing attention in recent years. Among them, OHRQoL was recommended as a proxy to measure patients’ well-being [1]. Many studies reported that apart from oral health, sociodemographic factors could also affect OHRQoL. For example, females reported higher impacts of oral health on quality of life than males did, and mother’s education level could influence their children’s OHRQoL [2–4]. When conducting studies in this area, all these factors should be taken into consideration [3, 5]. In addition, because psychosocial status is liable to change with age, quality of life is also a “dynamic construct” that is likely to change overtime [6]. Due to methodological difficulties, most studies were designed in a cross-sectional setting [3, 7–12]. Systematic reviews suggested cohort studies with population-based samples should be conducted to provide more evidence in this area [13–16].

This article presents a 6-year cohort study that aimed to analyse factors that may affect OHRQoL. The representative sample was first selected from 12-year-old students in Hong Kong. Then the subjects were followed up at ages 15 and 18. Cross-sectional analyses of this study have been published in previous articles [2, 4, 17].

## Methods

### Ethics, consent and permissions

The ethical approval of this study was granted by the Institutional Review Board of the University of Hong Kong/Hospital Authority Hong Kong West Cluster (UW 09–453). A written consent from parents/primary caregivers and a verbal consent from students were obtained when the students were under 18 years old. A written consent from students was obtained when they were 18 years old.

### Sampling method

Surveys of this cohort study were conducted in 2010, 2013 and 2015. Cluster randomized trial was used in this study. The sampling frame was all local secondary schools in Hong Kong (by law all children are required to attend secondary school). A random sample of 45 schools (approximately 10% of all local secondary schools) was selected from 18 districts in Hong Kong, SAR. Students born between April 1st and May 31st, 1997 were invited to participate in the oral health survey conducted by Faculty of Dentistry, The University of Hong Kong. The sample of this study was selected from the birth cohort of “children of 1997” [18]. Systematic health information, dental treatment history, ecosocial factors including father’s education, mother’s education, and household income were collected from a self-completed questionnaire. Subjects were excluded from the final analysis if they were systemically unhealthy, had orthodontic treatment history, or had oral diseases other than caries, periodontitis and malocclusion. Missing data in questionnaires was filled with the mode of the corresponding category.

Sample size was calculated based on previous studies [19–21]. The prevalence of orthodontic treatment need (ICON) was 80.3%; the mean CPQ scores (SD) were respectively 20.1 (14.0) and 14.8 (15.0) for “with treatment need” group and “without treatment need” group; α = 0.05, and 1-β = 0.8. With a lost rate of 30% at each follow-up and the design effect for cluster sampling considered, the sample sizes at ages 12, 15, and 18 should be 237, 166, and 116, respectively.

### Assessment of OHRQoL

For children aged 11 to 14 years old, the questionnaire of Child Perception Questionnaire (CPQ_11–14_) has been widely validated and used to measure OHRQoL [22, 23]. The self-completed questionnaire consists of 37 items namely oral symptoms domain (6 items), functional limitations domain (9 items), emotional well-being domain (9 items) and social well-being domain (13 items). Each item has a 5-point response format ranging from 0 to 4. The item scores of each domain are added together to get a domain score, and the scores of four domains are added together to get the total CPQ_11–14_ score. Higher scores represent poorer quality of life. To facilitate its use in clinical settings and population-based surveys, CPQ_11–14_ was shortened to 16 and 8 items by item impact and stepwise regression methods. In this research, the Chinese version of CPQ_11–14_ with 8 items (CPQ_11–14_-ISF: 8) and the Chinese version of CPQ_11–14_ with 37 items were used for 12-year olds and 15-year olds, respectively [23–25].

Oral health impact profile (OHIP) is a well-validated questionnaire that was developed in 1994 for adults. It was first designed as a self-completed questionnaire with 49 items (OHIP-49) [26] and was subsequently shortened to 14 items (OHIP-14) [27, 28]. The following 7 dimensions of oral health impact are measured: functional limitation, physical pain, psychological discomfort, physical disability, psychological disability, social disability, and handicap. For each question people are asked how frequently they had experienced the impact in the preceding 12 months. The responses followed a Likert-type scale coded as follows: ‘never’ = 0; ‘hardly ever’ = 1; ‘occasionally’ = 2; ‘fairly often’ = 3; and ‘very often’ = 4. The total score also can be calculated as the sum of the item scores, generating scores from 0 to 196 for OHIP-49 and from 0 to 56 for OHIP-14, with a higher score indicating more negative impacts and a lower OHRQoL. The Chinese version of OHIP-14 was used for 18-year olds in this study [28].

### Oral health examination

Community Periodontal Index (CPI) and the Decayed, Missing and Filled Teeth (DMFT) were used to measure periodontal and caries conditions according to the criteria of WHO [29]. Significant Caries Index (SiC index) was also used to classify caries; one third of the population with the highest caries score is selected and the mean DMFT for this subgroup constitutes the SiC Index value [30].

Index of Orthodontic Treatment Need (IOTN) with aesthetic component (AC) and dental health component (DHC), Dental Aesthetic Index (DAI), Index of Complexity, Outcome and Need (ICON), and Peer Assessment Rating (PAR) were used to assess orthodontic treatment need and complexity [31–36]. The classification methods have been published in previous articles [2, 4, 17].

Oral examination was performed using an intra-oral disposable mouth mirror with built-in LED light source and a CPI probe recommended by WHO [29]. A trained and calibrated dentist performed the oral examination according to the criteria of WHO [29]. The examiner of year 2010 and 2013 was different from that of 2015. Front-view dental photos were taken by extracting lips using oral retractors. These photos were used to assess IOTN (AC) by the examiner of 2015. Dental impressions were collected and the plaster models were sent to OrthoLab (Poland) to make digital models. Software O3DM (version3.8.5 (c) by OrthoLab, Poland) was used to analyse malocclusion on digital models by the examiner of 2015. Reassessments were performed among 10% randomly selected samples after 2 weeks of first assessment to test intra-examiner’s reliability.

### Statistical methods

Intra-examiner reliability was tested by kappa values for CPI, weighted kappa for IOTN (DHC) and IOTN (AC), and intra-class correlation coefficient (ICC) for DMFT, DAI score, and ICON score. Inter-examiner reliabilities for CPI and DMFT were tested by kappa value and ICC, respectively.

Longitudinal changes of OHRQoL and malocclusion were analysed with Wilcoxon signed ranks test for two-related samples, and Friedman’s test for three-related samples.

The effects of the factors on OHRQoL were analysed using generalized estimating equations (GEE) (model: Poisson loglinear). All subjects who participated in this study at age 12, 15 and 18 were entered into GEE analysis. OHRQoL scores were grouped into four ranks with quartile values as cut-offs; a higher rank represented a worse OHRQoL. Then the dependent variable was set as the ranks of OHRQoL. Independent variables were set as age, gender, father’s education, mother’s education, household income, periodontal status, caries experience, and orthodontic treatment needs. To avoid interaction effect, orthodontic treatment needs measured by different orthodontic indices were entered into GEE analysis separately. Both unadjusted and adjusted risk ratios (RR) were calculated.

## Results

Eligible students participating in surveys in 2010, 2013 and 2015 were 589 (305 females, 284 males), 364 (186 females, 178 males) and 300 (165 females, 135 males), respectively. Among these students, 331 (172 females, 159 males) participated in both surveys of 2010 and 2013, 204 (114 females, 90 males) participated in both surveys of 2013 and 2015, 276 (154 females, 122 males) participated in both surveys of 2010 and 2015, and 188 (106 females, 82 males) participated in all three surveys.

Missing data was found in questions related to family information and OHRQoL. The 12-year-old survey had the most missing data, with 25 subjects leaving one or two questions unanswered. Missing data were filled with the mode of the corresponding question.

For intra-examiner reliability tests, kappa values for CPI in 2010, 2013, and 2015 were respectively 0.740, 0.789, and 0.713; weighted kappa for IOTN (DHC) and IOTN (AC) were 0.918 and 0.790; ICC for DMFT in 2010, 2013, and 2015 were 0.990, 0.991, and 0.996, respectively; ICC for the scores of DAI and ICON were 0.821 and 0.820. For inter-examiner reliability test, kappa value for CPI was 0.660; ICC for DMFT was 0.986.

The oral health status of participants is shown in Table 1. The incidence of oral diseases increased slightly across the three surveys. Prevalence of unhealthy periodontal conditions was higher than that of caries. At least 85% of subjects had CPI scores above 0, whereas less than 60% of subjects had DMFT above 0 in the three surveys.

**Table 1:** Descriptive statistics of oral health status of participants

	12 years old		15 years old		18 years old	
	N	Percentage	N	Percentage	N	Percentage
**IOTN (DHC) treatment need**						
No need	321	54.50%	194	53.30%	161	53.67%
Borderline need	106	18.00%	78	21.43%	63	21.00%
Definite need	162	27.50%	92	25.27%	76	25.33%
**IOTN (AC) treatment need**						
No need	469	79.63%	290	79.67%	241	80.33%
Borderline need	89	15.11%	50	13.74%	38	12.67%
Definite need	31	5.26%	24	6.59%	21	7.00%
**DAI severity and treatment need**						
Normal or minor malocclusion-no treatment need or slight need	312	52.97%	153	42.03%	129	43.00%
Definite malocclusion-treatment selective	143	24.28%	111	30.49%	92	30.67%
Severe malocclusion-treatment highly desirable	87	14.77%	65	17.86%	45	15.00%
Very severe (handicapping) malocclusion-treatment mandatory	47	7.98%	35	9.62%	34	11.33%
**ICON treatment need**						
No	383	65.03%	241	66.21%	197	65.67%
Yes	206	34.97%	123	33.79%	103	34.33%
**ICON complexity**						
Easy	173	29.37%	103	28.30%	89	29.67%
Mild	292	49.58%	188	51.65%	147	49.00%
Moderate	67	11.38%	34	9.34%	29	9.67%
Difficult	33	5.60%	21	5.77%	22	7.33%
Very difficult	24	4.07%	18	4.95%	13	4.33%
**PAR**						
Almost ideal occlusion	122	20.71%	69	18.96%	71	23.67%
Acceptable occlusion	254	43.12%	119	32.69%	91	30.33%
Malocclusion	213	36.16%	176	48.35%	138	46.00%
**Periodontal status**						
CPI score = 0	80	13.58%	27	7.42%	16	5.33%
CPI score > 0	509	86.42%	337	92.58%	284	94.67%
**Caries experience**						
< SiC Index value	499	84.72%	317	87.09%	257	85.67%
> =SiC Index value	90	15.28%	47	12.91%	43	14.33%
DMFT = 0	403	68.42%	172	47.25%	123	41.00%
DMFT> 0	186	31.58%	192	52.75%	177	59.00%
**DMFT**		**Mean (SD)**		**Mean (SD)**		**Mean (SD)**
	589	0.57 (1.024)	364	1.70 (2.377)	300	1.92 (2.373)

*IOTN* Index of Orthodontic Treatment Need, *DHC* dental health component, *AC* aesthetic component, *DAI* dental aesthetic index, *ICON* Index of Complexity, Outcome and Need, *PAR* Peer Assessment Rating, *CPI* Community Periodontal Index, *SiC Index* Significant Caries Index, *DMFT* Decayed, Missing and Filled Teeth, *SD* standard deviation

SiC index value (SD) for 2010, 2013, and 2015 were 1.68 (1.115), 4.48 (2.242), and 4.72 (2.021), respectively

Longitudinal changes of OHRQoL and malocclusion are presented in Tables 2, 3 and 4. Subjects’ OHRQoL changed over time (*p* = 0.005, Table 2). Age 15 had a higher OHRQoL score than ages 12 and 18. IOTN (AC), DAI, and PAR detected an age-related change of malocclusion. Compared with age 12, age 15 and age 18 showed more severe levels of malocclusion. This result was confirmed by the comparisons between each two age periods: when compared with age 18, age 15 showed the same level of malocclusion (*p* > 0.05, Table 3), while age 12 showed a less severe level of malocclusion (*p* = 0.000 and 0.022 for DAI and PAR, respectively, Table 4).

**Table 2:** Longitudinal changes of 12 to 18 years old

	12 years old		15 years old		18 years old		
	Mean (SD)	Median (IQR)	Mean (SD)	Median (IQR)	Mean (SD)	Median (IQR)	***P*** value
**OHRQoL**	2.18(1.093)	2.00(2)	2.47(1.125)	2.00(3)	2.27(1.195)	2.00(2)	0.005**
**IOTN (DHC)**	1.65(0.829)	1.00(1)	1.71(0.824)	1.00(1)	1.69(0.822)	1.00(1)	0.375
**IOTN (AC)**	1.23(0.555)	1.00(0)	1.26(0.577)	1.00(0)	1.27(0.590)	1.00(0)	0.013*
**DAI**	1.65(0.880)	1.00(1)	1.95(1.004)	2.00(2)	1.93(0.987)	2.00(2)	0.000**
**ICON**	1.31(0.465)	1.00(1)	1.33(0.471)	1.00(1)	1.34(0.473)	1.00(1)	0.582
**ICON complexity**	2.03(0.961)	2.00(1)	2.05(1.015)	2.00(1)	2.07(1.029)	2.00(1)	0.386
**PAR**	2.14(0.706)	2.00(1)	2.27(0.790)	2.00(1)	2.27(0.798)	2.00(1)	0.000**

*OHRQoL* Oral Health-Related Quality of Life, *IOTN* Index of Orthodontic Treatment Need, *DHC* dental health component, *AC* aesthetic component, *DAI* dental aesthetic index, *ICON* Index of Complexity, Outcome and Need, *PAR* Peer Assessment Rating, *SD* standard deviation, *IQR* interquartile range; **: *p* < 0.01; *: *p* < 0.05

Sample size: 188; Statistical method: Friedman’s 2-way ANOVA by ranks (k samples): all pairwise multiple comparisons. OHRQoL were classified into quartiles; IOTN (DHC) and IOTN (AC): no need, borderline need, definite need; DAI: normal or minor malocclusion, definite malocclusion, severe malocclusion, very severe (handicapping) malocclusion; ICON: no, yes; ICON complexity: easy, mild, moderate, difficult, very difficult; PAR: almost ideal occlusion, acceptable occlusion, malocclusion

**Table 3:** The comparison of age 15 and age 18

	15 years old		18 years old		
	Mean (SD)	Median (IQR)	Mean (SD)	Median (IQR)	***p*** value
**OHRQoL**	2.47(1.129)	2.00(3)	2.31(1.186)	2.00(2)	0.129
**IOTN (DHC)**	1.71(0.830)	1.00(1)	1.68(0.825)	1.00(1)	0.295
**IOTN (AC)**	1.27(0.580)	1.00(0)	1.28(0.592)	1.00(0)	0.317
**DAI**	1.94(1.006)	2.00(2)	1.92(0.989)	2.00(1)	0.586
**ICON**	1.34(0.476)	1.00(1)	1.34(0.476)	1.00(1)	1.000
**ICON complexity**	2.07(1.026)	2.00(1)	2.09(1.042)	2.00(1)	0.513
**PAR**	2.26(0.793)	2.00(1)	2.26(0.799)	2.00(1)	1.000

*OHRQoL* Oral Health-Related Quality of Life, *IOTN* Index of Orthodontic Treatment Need, *DHC* dental health component, *AC* aesthetic component, *DAI* dental aesthetic index, *ICON* Index of Complexity, Outcome and Need, *PAR* Peer Assessment Rating, *SD* standard deviation, *IQR* interquartile range

Sample size: 204; two-related-samples test: Wilcoxon Signed Ranks Test. OHRQoL were classified into quartiles; IOTN (DHC) and IOTN (AC): no need, borderline need, definite need; DAI: normal or minor malocclusion, definite malocclusion, severe malocclusion, very severe (handicapping) malocclusion; ICON: no, yes; ICON complexity: easy, mild, moderate, difficult, very difficult; PAR: almost ideal occlusion, acceptable occlusion, malocclusion

**Table 4:** The comparison of age 12 and age 18

	12 years old		18 years old		
	Mean (SD)	Median (IQR)	Mean (SD)	Median (IQR)	***p*** value
**OHRQoL**	2.23(1.090)	2.00(2)	2.34(1.203)	2.00(2)	0.149
**IOTN (DHC)**	1.70(0.849)	1.00(2)	1.71(0.836)	1.00(1)	0.640
**IOTN (AC)**	1.23(0.537)	1.00(0)	1.26(0.576)	1.00(0)	0.059
**DAI**	1.69(0.897)	1.00(1)	1.95(1.010)	2.00(2)	0.000**
**ICON**	1.32(0.468)	1.00(1)	1.34(0.474)	1.00(1)	0.516
**ICON complexity**	2.01(0.961)	2.00(1)	2.06(1.023)	2.00(1)	0.129
**PAR**	2.14(0.710)	2.00(1)	2.22(0.809)	2.00(1)	0.022*

*OHRQoL* Oral Health-Related Quality of Life, *IOTN* Index of Orthodontic Treatment Need, *DHC* dental health component, *AC* aesthetic component, *DAI* dental aesthetic index, *ICON* Index of Complexity, Outcome and Need, *PAR* Peer Assessment Rating, *SD* standard deviation, *IQR* interquartile range; **: *p* < 0.01; *: *p* < 0.05

Sample size: 276; two-related-samples test: Wilcoxon Signed Ranks Test. OHRQoL were classified into quartiles; IOTN (DHC) and IOTN (AC): no need, borderline need, definite need; DAI: normal or minor malocclusion, definite malocclusion, severe malocclusion, very severe (handicapping) malocclusion; ICON: no, yes; ICON complexity: easy, mild, moderate, difficult, very difficult; PAR: almost ideal occlusion, acceptable occlusion, malocclusion

The results of GEE are presented in Table 5. Gender, parents’ education, household income, and caries experience did not affect OHRQoL, while age, malocclusion, and periodontal status showed an effect on OHRQoL. Compared with age 12, subjects had a higher risk of having worse OHRQoL at age 15 (adjusted RR = 1.06, 95%CI = 1.01–1.12, *p* = 0.032). Nevertheless, no difference was detected between age 12 and age 18 (*p* = 0.759). Only severe malocclusions had significant influence on OHRQoL. The more severe the malocclusion, the much higher the effect. Take DAI for example. While the adjusted RR was not significant for the “definite malocclusion” group (*p* = 0.074), the adjusted RR was 1.09 for the “severe malocclusion” group (*p* = 0.032), and it was increased into 1.17 for the “very severe malocclusion” group (*p* = 0.001).

**Table 5:** Generalized estimating equations between the factors and the OHRQoL from 12 to 18 years old

	Total OHRQoL			
	Unadjusted RR (95%CI)	P_**1**_	Adjusted RR (95%CI)	P_**2**_
**Sociodemographic status**				
**Age**				
12^a^				
15	1.07 (1.01, 1.12)	0.016*	1.06 (1.01, 1.12)	0.032*
18	1.02 (0.96, 1.09)	0.479	1.01 (0.95, 1.08)	0.759
**Gender**				
F^a^				
M	0.98 (0.92, 1.04)	0.485	0.98 (0.92, 1.04)	0.510
**Father’s education**				
Primary school graduate or below^a^				
Secondary school graduate or below	0.99 (0.91, 1.07)	0.713	1.02 (0.93, 1.11)	0.703
College graduate or above	0.94 (0.84, 1.04)	0.239	0.99 (0.88, 1.12)	0.929
**Mother’s education**				
Primary school graduate or below^a^				
Secondary school graduate or below	0.92 (0.85, 1.00)	0.062	0.93 (0.85, 1.02)	0.108
College graduate or above	0.89 (0.79, 0.99)	0.035*	0.94 (0.82, 1.08)	0.362
**Household income**				
below HK$10,000^a^				
HK$10,001-HK$20,000	1.02 (0.95, 1.10)	0.619	1.02 (0.94, 1.10)	0.646
HK$20,001-HK$30,000	1.00 (0.91, 1.09)	0.926	1.00 (0.91, 1.09)	0.987
HK$30,001-HK$40,000	1.03 (0.93, 1.13)	0.602	1.03 (0.93, 1.14)	0.552
Over HK$40,001	0.92 (0.83, 1.02)	0.103	0.93 (0.84, 1.04)	0.229
**Periodontal and caries status**				
**Periodontal status**				
CPI score = 0^a^				
CPI score > 0	1.16 (1.05, 1.27)	0.002**	1.14 (1.04, 1.25)	0.007**
CPI score < 2^a^				
CPI score > =2	1.12 (1.05, 1.19)	0.000**	1.11 (1.04, 1.18)	0.002**
**Caries experience**				
DMFT = 0^a^				
DMFT> 0	1.01 (0.96, 1.07)	0.696	0.99 (0.93, 1.04)	0.621
DMFT<SiC value^a^				
DMFT> = SiC value	1.03 (0.95, 1.11)	0.480	1.02 (0.94, 1.10)	0.652
**Malocclusion**				
**IOTN (DHC) treatment need**				
No need^a^				
Borderline need	1.08 (1.00, 1.16)	0.046*	1.07 (0.99, 1.15)	0.094
Definite need	1.09 (1.02, 1.17)	0.016*	1.09 (1.01, 1.16)	0.019*
**IOTN (AC) treatment need**				
No need^a^				
Borderline need	1.12 (1.03, 1.21)	0.006**	1.11 (1.03, 1.20)	0.007**
Definite need	1.07 (0.94, 1.22)	0.319	1.07 (0.94, 1.21)	0.312
No need^a^				
Borderline and definite need	1.10 (1.03, 1.19)	0.007**	1.10 (1.03, 1.18)	0.007**
**DAI severity and treatment need**				
Normal or minor malocclusion-no treatment need or slight need^a^				
Definite malocclusion-treatment selective	1.06 (1.00, 1.13)	0.055	1.06 (0.99, 1.13)	0.074
Severe malocclusion-treatment highly desirable	1.10 (1.02, 1.19)	0.014*	1.09 (1.01, 1.18)	0.032*
Very severe (handicapping) malocclusion-treatment mandatory	1.18 (1.07, 1.29)	0.001**	1.17 (1.07, 1.28)	0.001**
**ICON treatment need**				
No^a^				
Yes	1.10 (1.03, 1.16)	0.003**	1.10 (1.03, 1.16)	0.003**
**ICON complexity**				
Easy^a^				
Mild	1.06 (0.99, 1.14)	0.106	1.05 (0.98, 1.13)	0.157
Moderate	1.12 (1.01, 1.25)	0.038*	1.12 (1.01, 1.25)	0.037*
Difficult	1.17 (1.03, 1.32)	0.014*	1.16 (1.03, 1.30)	0.018*
Very difficult	1.17 (1.01, 1.35)	0.038*	1.17 (1.01, 1.34)	0.031*
**PAR score range**				
Almost ideal occlusion^a^				
Acceptable occlusion	1.01 (0.93, 1.09)	0.857	1.01 (0.93, 1.09)	0.906
Malocclusion	1.10 (1.01, 1.19)	0.023*	1.08 (1.00, 1.17)	0.051
Almost ideal or acceptable occlusion^a^				
Malocclusion	1.09 (1.03, 1.16)	0.003**	1.08 (1.02, 1.15)	0.011*

*OHRQoL* Oral Health-Related Quality of Life, *RR* risk ratio, *CI* confidence interval, *F* female, *M* male, *CPI* Community Periodontal Index, *DMFT* Decayed, Missing and Filled Teeth, *SiC index* Significant Caries Index, *IOTN* Index of Orthodontic Treatment Need, *DHC* dental health component, *AC* aesthetic component, *DAI* dental aesthetic index, *ICON* Index of Complexity, Outcome and Need; *PAR* Peer Assessment Rating

Total sample size: 1253. Statistical method: Generalized estimating equations (subject variables: students’ reference number; within-subject variables: age; covariance matrix: robust estimator; correlation structure: unstructured; type of model: Poisson loglinear; model: main effects), each orthodontic index adopted one separate regression; dependent variable: total CPQ score of 12 and 15 years old and OHIP score of 18 years old classified into four groups with cut-off points as quartile (1: scores<=first quartile; 2: first quartile<scores<=second quartile; 3: second quartile<scores<= third quartile; 4: scores > third quartile); a: reference group; **: *P* < 0.01; *: *P* < 0.05

Adjusted RR: malocclusions adjusted for age, gender, father’s education level (primary school graduate or below; secondary school, post-secondary or above), mother’s education level (levels set as father’s education), household income (below HK$10000, HK$10001-HK$20000, HK$20001-HK$30000, HK$30001-HK$40000, HK$40001 or above), caries experience (DMFT = 0, DMFT> 0), and periodontal status (CPI score = 0, CPI score > 0); gender, socioeconomic status, periodontal and caries status adjusted for the precious variables and malocclusion measured by ICON treatment need (no, yes)

Unhealthy periodontal conditions had an adverse effect on OHRQoL. For example, subjects with CPI scores above 0 were 1.14 times as likely to have a worse OHRQoL when compared to subjects with CPI scores equal to 0 (*p* = 0.007).

## Discussion

This cohort study investigated the influence factors of OHRQoL. Subjects were randomly selected from Hong Kong at age 12 and were followed up at age 15 and age 18. Malocclusion had worsened from age 12 to 15, but maintained stable from age 15 to 18. Gender, parents’ education, household income, and caries experience did not affect OHRQoL. Subjects had worse OHRQoL at age 15 than at age 12 or 18. Unhealthy periodontal status was more prevalent than caries in this cohort, and it affected OHRQoL negatively. Severe malocclusions showed negative influence on OHRQoL as well. A more severe level of malocclusion was associated with a higher effect on OHRQoL.

In this study, CPQ_11–14_ was used at ages 12 and 15, and OHIP-14 was used at age 18. These questionnaires have different subscales; hence, only the total scores of these questionnaires could be analysed. However, these total scores could not be compared directly because they had different score ranges. Therefore, these scores were classified into four ranks using quartile values as cut-offs. In this way the ranks of OHRQoL could be compared using GEE.

When analysing the longitudinal changes of malocclusion, IOTN (DHC) and ICON detected no change through age 12 to 18, while IOTN (AC), DAI and PAR detected an ascending of malocclusion severity from age 12 to 15. This indicated orthodontic indices had different response to the change of malocclusion. Different indices have different calculation methods for malocclusion. When certain changes of malocclusion remained in the same classification in one index, it might show a change in another. This study demonstrated when IOTN (AC), DAI and PAR are used to study age-related influence of malocclusion on OHRQoL from age 12 to 15, it is necessary to assess malocclusion at each follow-up.

Unhealthy periodontal conditions were more prevalent than caries through all three surveys of this study. There may be two possible explanations. First, caries is effectively prevented by water fluoridation in Hong Kong. Second, children are more susceptible to gingivitis in puberty period [37]. Hong Kong government has put great efforts in caries prevention; the School Dental Care Service provides dental care to all primary school students. As a result, most subjects in this study (68.42%) had no caries at age 12. For those who had caries, the cavities were either small/shallow, or filled, which would not cause pain; only a few subjects showed severe caries that was untreated. Therefore, caries did not affect subjects’ OHRQoL in this study. When cross-sectional analysis was performed at each age, the effect was only found in the domain of social well-being at age 12 [2]. The adverse effect of unhealthy periodontal conditions on OHRQoL was detected by this study. This result showed proper oral health promotion for periodontitis should be conducted to reduce the unhealthy periodontal conditions in subjects from age 12 to 18 in Hong Kong.

There was a fluctuation of subjects’ OHRQoL from age 12 to 18, showing deterioration at age 15, and improvement at age 18. This result was supported by the findings of our systematic review: subjects’ OHRQoL were less likely to be impacted by malocclusion in age 15–18 than in age 12–15 [13]. Possible explanation was that subjects’ physical and psychological statuses changed dramatically in the puberty period; they became more aware of their appearance, and their psychological status was relatively more vulnerable and changeable [38, 39]; later their view of themselves and of the outside world tended to be more stable [40, 41]. This result indicated clinicians should pay more attention when they intend to improve patients’ OHRQoL at age 15.

In this analysis, sociodemographic factors did not have significant influence on OHRQoL. However, cross-sectional analyses in previous studies indicated some effects of sociodemographic factors on OHRQoL, with gender showing an effect at ages 12 and 15, parents’ education showing an effect at ages 12 and 18, and household income showing an effect at age 18 [2, 4, 17].

Studies reported that periodontitis and temporomandibular disorders are more likely to occur in subjects with more severe malocclusion than in subjects with less severe or no malocclusion [42, 43]. This conclusion seems to be supported by this study. Only severe malocclusions showed negative effects on OHRQoL in this study. Children with more severe malocclusion were more likely to report oral symptoms and negative emotional experiences in OHRQoL questionnaire. These results are also echoed by our cross-sectional analyses and systematic reviews [2, 4, 13, 14, 17]. Orthodontic treatment for children with severe malocclusion might improve both their oral symptoms and emotional experiences.

All orthodontic indices were capable of detecting the influence of malocclusion on OHRQoL in this study. Moreover, all indices detected a gradient ascending of the RR value across the levels of malocclusion, except for IOTN (AC). IOTN (AC) only reflects the dental aesthetics in anterior dental arches; no inter- or intra- arch malocclusion is considered [44]. Dental aesthetics was associated with people’s social attractiveness. In the cross-sectional analysis at age 12, IOTN (AC) showed a better ability to detect the effect of malocclusion on the domain of social well-being [2].

This cohort study may provide some evidence for the influence factors of OHRQoL. However, the subjects were exclusively sampled in Hong Kong. When generalizing the conclusion to other regions, differences in ethnicity, geography, culture, and economics need to be considered.

## Conclusions

This cohort study focused on the influence factors of OHRQoL in a representative sample. Severity of malocclusion increased from age 12 to 15 but remained stable from age 15 to 18. Subjects were likely to have a worse OHRQoL at age 15 than at ages 12 and 18. In this cohort, socioeconomic factors did not affect OHRQoL. Unhealthy periodontal conditions were more prevalent than caries; furthermore, it was periodontal status, but not caries, that had a negative effect on OHRQoL. Severe malocclusions also had negative effects on OHRQoL; a more severe level of malocclusion was associated with a higher risk of worse OHRQoL.

## Data Availability

The datasets used and/or analysed during the current study are available from the corresponding author on reasonable request.

## Abbreviations

AC: Aesthetic component
CI: Confidence interval
CPI: Community Periodontal Index
CPQ: Child Perceptions Questionnaire
DAI: Dental aesthetic index
DHC: Dental health component
DMFT: Decayed, Missing and Filled Teeth
GEE: Generalized estimating eqs.
ICC: Intra-class correlation coefficient
ICON: Index of Complexity, Outcome and Need
IOTN: Index of Orthodontic Treatment Need
IQR: Interquartile range
ISF: Short-form questionnaire generated by item impact method
OHIP: Oral Health Impact Profile
OHRQoL: Oral Health-Related Quality of Life
PAR: Peer Assessment Rating
RR: Risk ratio
SD: Standard deviation
SiC index: Significant Caries Index
WHO: World Health Organization
